# Parenthood in a Swedish prospective cohort of 1,378 adolescents and young adults banking semen for fertility preservation at time of cancer diagnosis

**DOI:** 10.3389/fendo.2024.1502479

**Published:** 2024-12-10

**Authors:** Kristina Weibring, Frida E. Lundberg, Gabriella Cohn-Cedermark, Kenny Alexandra Rodriguez-Wallberg

**Affiliations:** ^1^ Department of Oncology-Pathology, Karolinska Institutet, Stockholm, Sweden; ^2^ Department of Oncology, Karolinska University Hospital, Stockholm, Sweden; ^3^ Department of Medical Epidemiology and Biostatistics, Karolinska Institutet, Stockholm, Sweden; ^4^ Laboratory of Translational Fertility Preservation, Karolinska Institutet, Stockholm, Sweden; ^5^ Department of Reproductive Medicine, Division of Gynecology and Reproduction, Karolinska University Hospital, Stockholm, Sweden

**Keywords:** fertility preservation, cancer, AYAs, cryopreservation, reproductive outcome, oncofertility

## Abstract

**Background:**

The possibility of future parenthood is a highly relevant issue for patients of reproductive age facing oncologic treatment. This study aimed to investigate how fatherhood was achieved in a patient cohort of adolescents and young adults (AYAs) banking semen at time of cancer diagnosis and to determine the effectiveness of cryopreservation aimed at fertility preservation in the cohort.

**Materials and methods:**

Observational cohort study examining AYAs with a cancer diagnosis who underwent semen banking for fertility preservation at Karolinska University Hospital 1988-2020, as part of the Stockholm regional fertility preservation program. This cohort is being prospectively followed since time of referral to the program, with most individuals included when presenting with primary cancers (Study Registration: ClinicalTrials.gov NTC04602962). Individuals achieving adulthood in the cohort were followed-up regarding their reproductive outcomes by linking to the Swedish Multi-generation Register, to identify fatherhood through natural conception or adoption, and to the Swedish National Quality Registry for Assisted Reproduction to identify parenthood through medical assistance, including the use of own sperm either cryopreserved or fresh, or donor sperm.

**Results:**

Of the 1,378 patients referred during the study period, 1,357 were eligible for fatherhood analysis (aged >20 years at the end of follow-up, December 31, 2021). In total, 493 men became fathers following cancer treatment: 399 (81%) did so naturally, 87 (18%) via assisted reproductive techniques (including two using donor sperm), and 7 (1%) through adoption. Of the 92 patients who used their cryopreserved sperm for assisted reproductive techniques, 34 (37%) successfully fathered a child. The patients may have had children prior to cryopreservation.

**Conclusion:**

A large proportion of AYA cancer survivors achieved fatherhood through natural conception in this cohort, whereas less than 1 in 5 survivors needed medical assistance to conceive. Although a low utilization rate of cryopreserved sperm was found in this cohort, its use was highly effective in the group that developed infertility. At present there are no standardized predictors of testicular toxicity after cancer treatment, and inter-individual variability is high. Further research is needed to identify patients at risk of infertility who would benefit from fertility preservation.

## 1 Introduction

Early detection and advancements in cancer treatments have improved survival rates, leading to a greater focus on improving patient´s quality of life (1). Fertility preservation is a highly relevant factor for cancer survivors (2, 3), as treatment-related fertility issues can cause emotional distress, strained relationships, and affect the overall health, in addition to the risk of unwanted sterility (4). A recent Swedish study of 1010 young cancer survivors found that out of the 316 male cancer patients in the study, 27% experienced high fertility-related distress 1.5 years after treatment (5). This underscores the need for comprehensive reproductive healthcare before and after treatment, especially for those who are single, childless, experiencing anxiety, and desire to have future children (6).

Following international guidelines (7–9), Sweden has established national guidelines for fertility preservation for children, adolescents and young adults (AYAs) with cancer (10). These guidelines are integrated into various diagnosis-specific healthcare programs (11, 12). Sperm cryopreservation is well-established and the standard method for fertility preservation feasible from around 12-13 years of age, at the onset of spermarche (13, 14). The method is straightforward and effective, even with less-than-ideal semen samples (15, 16). In addition to enabling future biological fatherhood, cryopreservation can enhance patients’ self-esteem and help them cope with cancer and its treatment (15, 17, 18).

For AYAs who have banked sperm post-puberty, success rates with cryopreserved sperm using assisted reproductive techniques like conventional *in vitro* fertilization (IVF) or intracytoplasmic sperm injection (ICSI) are generally high. IVF is typically used when sperm quality is normal or only slightly reduced, whereas ICSI is preferred for severe quality impairments or very low sperm counts (19, 20). Research has indicated that frozen sperm stored for over 40 years retains functionality after thawing (21).

This observational cohort study aimed to investigate how parenthood was achieved among cancer survivors who cryopreserved sperm at diagnosis.

Ethical permissions have been granted for all parts of this study (Dnr 2011/1758-31/2, amendments 2018/275-32 and 2018/2255/32).

## 2 Materials and methods

### 2.1 Population and study design

**Figure 1:**
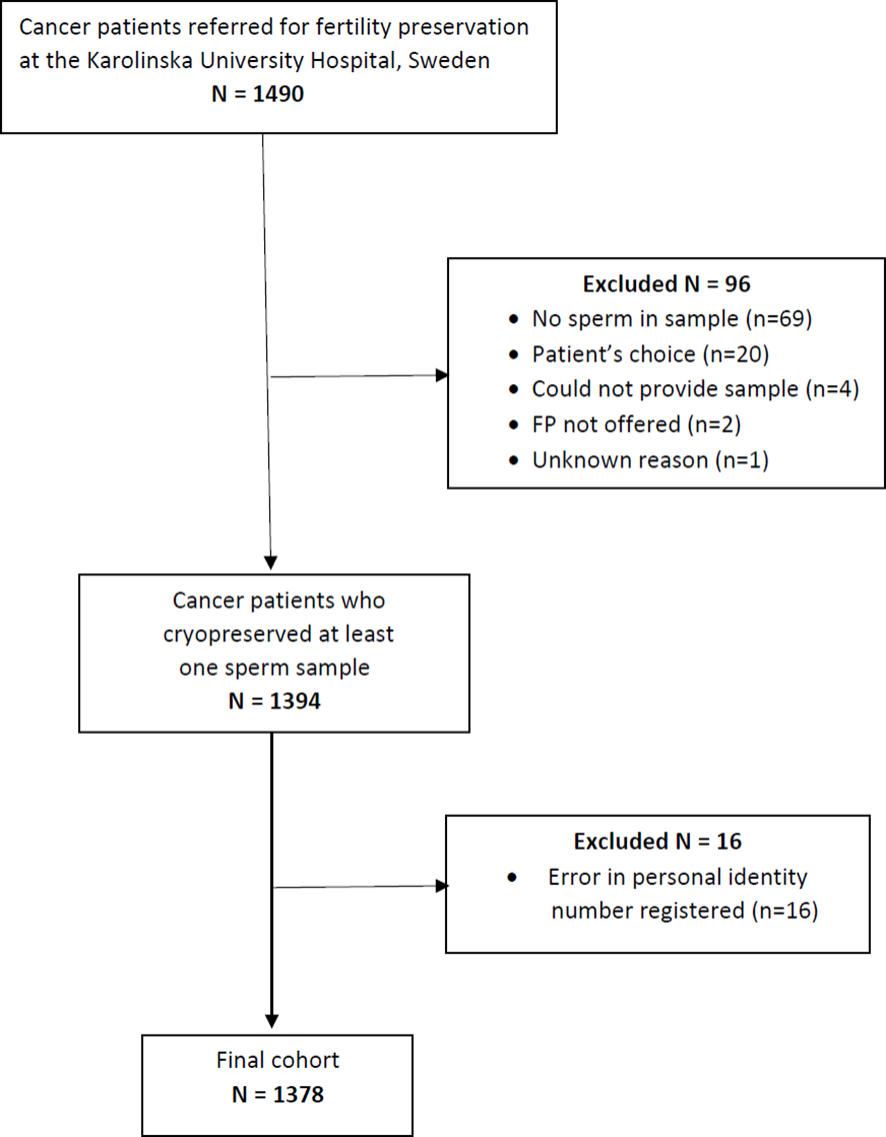
Flow chart of cohort.

Patients referred to the Stockholm regional service for fertility preservation at time of cancer diagnosis that attempted sperm cryopreservation between 1988 and 2020 were investigated. The regional program for fertility preservation is located at the Reproductive Medicine Clinic, Karolinska University Hospital, covering a healthcare region of around 2 Million inhabitants. Data on semen quality and cryopreservation were extracted from the clinical register. Out of 1490 AYA patients referred, 96 lacked semen samples, whereof 20 chose not to cryopreserve sperm, 69 lacked sperm in delivered sample, 4 could not produce a sample, 2 were not offered cryopreservation and 1 had not delivered any semen sample, for unknown reason. Follow-up for the entire cohort was conducted using the unique personal identification numbers assigned to all Swedish citizens. Cohort members were linked to data from national population-based registries maintained by Statistics Sweden, as well as the National Quality Register for Assisted Reproduction (Q-IVF). Linkage was possible for all but 16 patients where the personal identity number was not correctly registered, resulting in a study population of 1378 patients as shown in **Figure 1**.

We report on the proportion of patients who achieved parenthood through assisted reproduction with fresh or cryopreserved semen, natural conception, or adoption. The effectiveness of semen banking was evaluated as the proportion using cryopreserved semen in fertility treatments and the success rate of these treatments.

### 2.2 Sperm cryopreservation

Each patient provided at least one semen sample after a recommended abstinence period from sexual activity of 3 to 7 days, following current guidelines (22–24). Samples were analyzed and cryopreserved at the accredited laboratory for semen analysis, Karolinska University Hospital, Stockholm, Sweden. (ISO 9001:2015). Cryopreservation followed the WHO guidelines at the time, taking place after sperm preparation and before gonadotoxic treatment (22–24). While some patients with testicular cancer had undergone orchiectomy before cryopreservation, the majority provided semen samples prior to orchiectomy.

### 2.3 Outcomes

Information regarding the use of cryopreserved sperm was obtained from the treatment clinical registry of the Clinic of Reproductive Medicine, Karolinska University Hospital. Data on IVF/ICSI treatments and resulting births were obtained from Q-IVF for the years 2007 to 2021. Data on fatherhood before and during the follow-up period was sourced from the Swedish Multi-generation Register, which links individuals to their biological and adopted children. Information on country of birth and date of death was obtained from the Register of the Total Population, and emigration dates were obtained from the Migration Register. Each individual was followed from either age 20 or the date of cryopreservation, until the birth of their first child, adoption, emigration, death, or the end of follow-up (December 31st, 2021), whichever came first. In analyses of time to the first child after treatment, all men were followed from age 20 or the date of cryopreservation, whichever came last.

### 2.4 Statistical analysis

The return and utilization proportion were determined by dividing the number of cancer survivors returning for fertility counseling and treatment by the total number of individuals, aged 20 or older, residing in Sweden, who underwent semen banking and had sperm cryopreserved for at least one year. The crude probability of fathering a child (by any method except adoption) was calculated using the Kaplan Meier estimator and presented graphically stratified by age at cryopreservation. In these analyses, the crude probability is calculated among the men who are still alive (i.e. censoring at death). Cox proportional hazards models were used to estimate hazard ratios of fathering a child, with 95% confidence intervals. The adjusted model included calendar year and fatherhood at cryopreservation, and attained age as a time-varying covariate. The cumulative incidence of fathering a child in the presence of the competing risk of death was estimated non-parametrically using the command stcompet in Stata (25). Results are expressed as probabilities of fathering a child, or death due to any cause, after cryopreservation of sperm and presented stratified by cancer diagnosis and fatherhood status at cryopreservation. All analyses were performed using Stata statistical software version 18 (StataCorp).

## 3 Results

**Figure 2:**
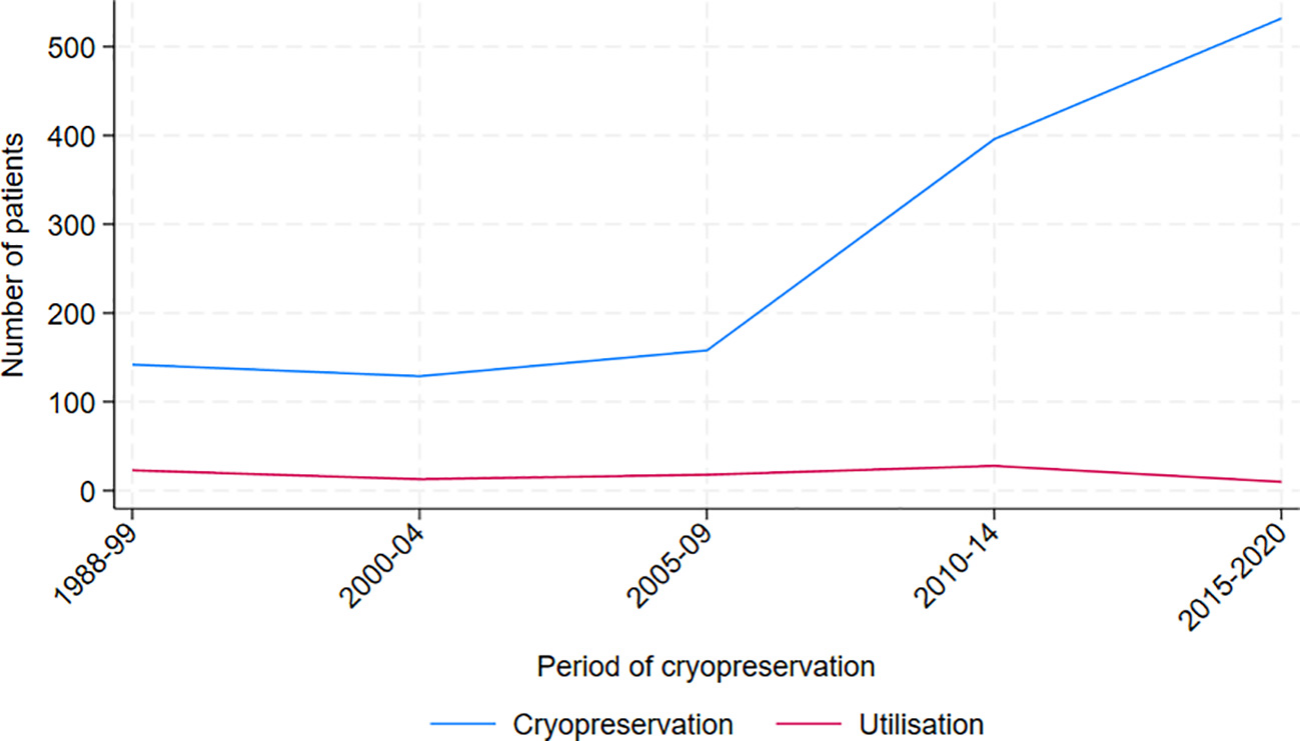
Total number of men cryopreserving and utilizing cryopreserved sperm, by period of cryopreservation.

Between 1988 and 2020, a total of 1,378 AYAs diagnosed with cancer underwent cryopreservation of sperm. The majority of the patients were between 30 and 39 years old, and the median age at cryopreservation in the cohort was 32 years (range 12 to 66 years). Testicular cancer, including seminoma (n=337, 24.5%) and non-seminoma (n=323, 23.4%), was the most prevalent diagnosis. (**Table 1**), followed by lymphoma including Hodgkin lymphoma (n= 143, 10.4%) and non-Hodgkin lymphoma (n=119, 8.6%). Cryopreservation rates increased over the study period, from less than 150 in the first 11 years (1988–1999) to 500 in the most recent five-year period (**Figure 2**).

**Table 1:** Description of cohort.

	Patients		Age at cryopreservation (years)	
	*N*	%	Median	Range
Total	1378	100.0	32	12-66
Diagnosis				
Testicular seminoma	337	24.5	33	18-59
Testicular non-seminoma	323	23.4	28	14-50
Hodgkin lymphoma	143	10.4	28	14-54
Non Hodgkin lymphoma	119	8.6	33	13-52
Acute leukemia	84	6.1	28	15-49
Other hematological malignancies*	46	3.3	37	18-53
Prostate	69	5.0	49	41-66
Colorectal	58	4.2	38	18-55
CNS	49	3.6	32	15-55
Sarcoma	46	3.3	20	12-46
Other solid tumors**	104	7.5	34	13-58
Calendar year at cryopreservation				
1988-1999	142	10.3	29	14-54
2000-2004	129	9.4	32	17-55
2005-2009	158	11.5	32	15-58
2010-2014	399	29.0	32	13-66
2015-2020	550	39.9	32	12-59
Country of birth				
Sweden	1128	81.9	31	12-66
Other	250	18.1	34	15-65
No of children before cryopreservation				
0	959	69.6	29	12-66
1	216	15.7	35	19-56
2	160	11.6	38	23-65
3 or more	43	3.1	45	30-55

*Other hematological tumors include chronic myeloid leukemia, myeloma and myelodysplastic syndrome.

**Other solid tumors include testicular cancer of unspecified type, malignant melanoma and tumors of the liver, digestive tract, urinary tract, head and neck.

Of the original cohort of 1,378 AYAs who cryopreserved sperm, seven individuals passed away before turning 20, and 14 had not yet reached that age by the end of follow-up in December 2021. Hence, 1,357 individuals were alive and 20 years or older at that time, thus suitable for our analyses of fatherhood. Among them, 493 (36%) had successfully become fathers.

The majority of men achieved fatherhood through natural conception (n=399, 81%), with most having been treated for testicular cancer (n=236, 59%). Among survivors of lymphoma, 75 patients (19%) were able to conceive naturally, while in the acute leukemia group, 14 patients (4%) succeeded (**Table 2**).

**Table 2:** Total number of men who achieved fatherhood, by method.

	Natural conception		Use of ART*								Adopted		Total
			Own sperm, frozen		Own sperm, fresh		Own sperm, uns		Donated sperm				
	N	%	N	%	N	%	N	%	N	%	N	%	N
Total	399	80.9	34	6.9	38	7.7	13	2.6	2	0.4	7	1.4	493
Diagnosis													
Testicular seminoma	121	88.3	2	1.5	9	6.6	4	2.9	0	0.0	1	0.7	137
Testicular non seminoma	115	83.3	10	7.2	9	6.5	3	2.2	0	0.0	1	0.7	138
Hodgkin lymphoma	40	72.7	5	9.1	6	10.9	3	5.5	0	0.0	1	1.8	55
Non Hodgkin lymphoma	35	74.5	4	8.5	4	8.5	1	2.1	1	2.1	2	4.3	47
Acute leukemia	14	70.0	2	10.0	2	10.0	1	5.0	1	5.0	0	0.0	20
Other hematological**	6	54.5	4	36.4	0	0.0	0	0.0	0	0.0	1	9.1	11
Prostate	8	66.7	2	16.7	1	8.3	1	8.3	0	0.0	0	0.0	12
Colorectal	13	86.7	1	6.7	1	6.7	0	0.0	0	0.0	0	0.0	15
Sarcoma	4	80.0	1	20.0	0	0.0	0	0.0	0	0.0	0	0.0	5
CNS	13	81.3	2	12.5	1	6.3	0	0.0	0	0.0	0	0.0	16
Other solid tumors***	30	81.1	1	2.7	5	13.5	0	0.0	0	0.0	1	2.7	37

*ART, Assisted Reproductive Technology.

**Other hematological include chronic myeloid leukemia, myeloma and myelodysplastic syndrome.

***Other solid tumors include testicular cancer of unspecified type, malignant melanoma and tumors of the lung, liver, digestive tract, head and neck.

Among the 1,357 survivors aged 20 or older by the end of follow-up in December 2021, 92 (6.8%) opted to use their cryopreserved sperm for IVF/ICSI. Of these 92, 34 (37%) successfully became fathers using their frozen sperm. Additionally, 38 men achieved fatherhood through IVF/ICSI using their fresh sperm and 13 by using own sperm (it is unclear whether frozen or fresh). Two men used donated sperm, and seven men chose adoption (**Table 2**). Hence, out of 493 patients succeeding in becoming fathers, 484 became biological fathers and 9 became non-biological fathers.

It is common practice that when a cancer survivor who has previously frozen sperm before cancer treatment seeks fertility treatment years later, the fertility work-up includes an evaluation of both the female factors and a request for the patient to provide a semen sample. If spermatogenesis has resumed and sperm are present in the sample, the patient is typically offered the option to proceed with treatment using fresh sperm. If no sperm are present, the frozen sperm is used instead. In cases where there is no frozen sperm available and no sperm production is occurring, donor sperm will be used.

The median age at the time of achieving fatherhood through natural conception or ART was 35 years (range 23-72) years (**Table 3**). Among the seven individuals who adopted a child, the median age at time of first adoption was 44 years (range 37-60 years).

**Table 3:** Median age at first biological child, by diagnosis.

	Fathers		Age at first child	
	*N*	%	Median	Range
Total	486	100.0	35	23-72
Diagnosis				
Testicular seminoma	136	28.0	35	26-47
Testicular non seminoma	137	28.2	33	23-47
Hodgkin lymphoma	54	11.1	34	24-45
Non Hodgkin lymphoma	45	9.3	34	27-47
Acute leukemia	20	4.1	32	26-51
Other hematological*	10	2.1	41	33-50
Prostate	12	2.5	55	42-72
Colorectal	15	3.1	39	30-52
CNS	16	3.3	36	31-50
Sarcoma	5	1.0	32	29-46
Other solid tumors**	36	7.4	37	25-49

*Other hematological tumors include chronic myeloid leukemia, myeloma and myelodysplastic syndrome.

**Other solid tumors include testicular cancer of unspecified type, malignant melanoma and tumors of the lung, digestive tract, head and neck.

### 3.1 Assisted reproductive outcomes

Data on various ART methods were retrieved from the Q-IVF register, which has been in use in Sweden since 2007. From that year onwards, 393 patients (32%) successfully achieved their first fatherhood following cryopreservation. Among them, 68 patients underwent ICSI, 10 underwent IVF and two utilized ICSI with donated sperm. Consequently, 78 patients (20%) experienced successful IVF/ICSI treatments from 2007 onwards, ICSI being the most successful method. Of these patients, 28 patients used their own frozen sperm, 37 used their own fresh sperm, and 13 used own sperm (whether frozen/fresh was unknown). Additionally, three patients pursued adoption while 312 patients conceived through natural conception (**Supplementary Table S1**).

### 3.2 Cumulative incidence of fatherhood and death

**Figure 3:**
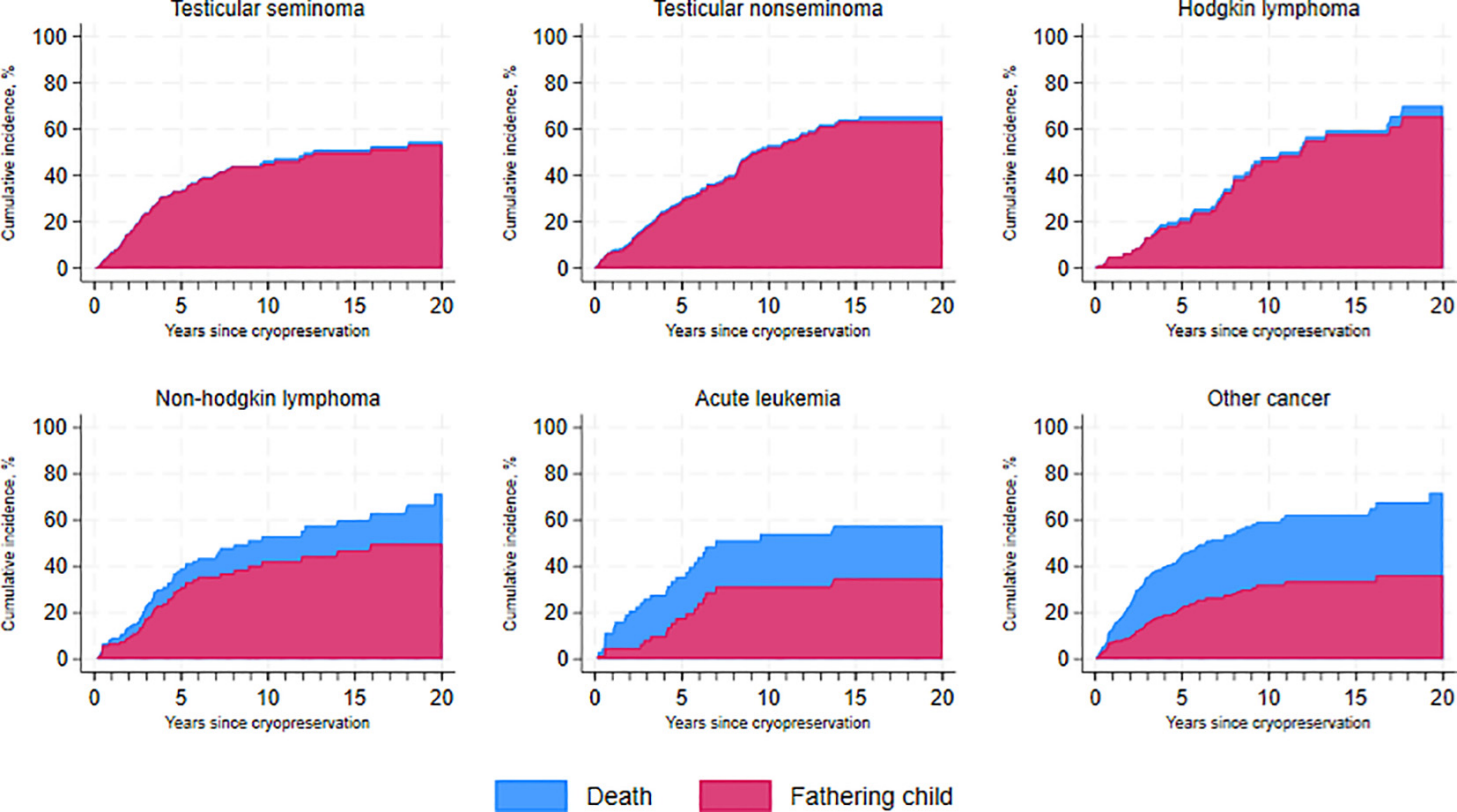
Cumulative incidence of fatherhood and death over time since cryopreservation, by cancer type.

**Figure 3** illustrates the cumulative incidence of fatherhood over time since cryopreservation across the entire cohort, accounting for the competing risk of death from any cause, and categorized by diagnosis type. As shown, patients with testicular cancer - both seminoma and non-seminoma – demonstrate very high survival rates compared to those with acute leukemia, where approximately 20% of the patients have passed away within the first five years after cryopreservation.

Our study further found that, 10 years after cryopreservation, 45% (95% CI, 0.38–0.51) of seminoma patients and 52% (95% CI, 0.45–0.59) of non-seminoma patients had become fathers, with the majority still alive 20 years after cryopreservation. Similar trends were observed in survivors of Hodgkin lymphoma (46%) and non-Hodgkin lymphoma (42%), becoming fathers 10 years post-cryopreservation. Survivors of acute leukemia had a cumulative incidence of fatherhood of 31% (95% CI, 0.19–0.44) at five years after cryopreservation.

Additionally, patients who already had children prior to cryopreservation were more likely to become fathers sooner than those without previous children (**Supplementary Figure S1**).

In univariable analysis, the fatherhood ratio was lower among men with prostate cancer (HR 0.43, 95% CI 0.23-0.76) and hematological malignancies other than acute leukemia (HR 0.47, 95% CI 0.25-0.89), compared to men with testicular seminoma (**Supplementary Table S2**). In analysis adjusted for calendar year at cryopreservation, having children at cryopreservation and attained age, there were no significant differences in fatherhood rates for any diagnosis type compared to men with testicular seminoma. Fatherhood rates were higher among men ages 30-39 compared to ages 20-29 (HR 2.54, 95% I 1.96-3.30), and men with previous children had slightly higher fatherhood rates (HR 1.23, 95% CI 1.00-1.52) compared to men without children.

### 3.3 Time to first child among those still alive

Among the men who were still alive, the probability of successfully fathering a child 5 years after cryopreservation, regardless of the method used, was approximately 25% for individuals in the 20-29 age group. This crude probability steadily increased over time, reaching around 70%, 15 years after cryopreservation. For individuals aged 30-39 years, the crude probability was approximately 40% five years post-cryopreservation, with a subsequent increase to about 60%, 15 years after cryopreservation (**Supplementary Figure S2**). Among those aged 40 years or older, 20% had a child 10 years after cryopreservation. The mean age to achieve fatherhood after fertility preservation was highest in men with prostate cancer (54.5 years) and lowest in the group with testicular non-seminoma (33.4 years).

## 4 Discussion

This study provides detailed data on how AYA cancer survivors, who were offered semen banking at time of cancer diagnosis from adolescence to adulthood, achieved fatherhood from 20 years of age and onwards, and how many of them who utilized their cryopreserved sperm.

Irrespective of the method used, 36% of the individuals in the cohort became fathers after cryopreservation and cancer treatment. Predominantly, these individuals were diagnosed with testicular cancer and lymphoma, reflecting the incidence rates of these diseases in young patients of reproductive age (26). While patients with testicular cancer represent the largest group for sperm cryopreservation, they are also a group that may regain natural fertility after cancer treatment (27). Our results, as demonstrated in **Table 2**, indicate that patients with testicular cancer and lymphoma have high success rates of natural conception after cancer treatment, at 59% and 19%, respectively. This suggests that many of these patients will maintain fertility post-treatment. However, most patients with testicular cancer undergo unilateral orchiectomy, which can pose a threat to future fertility. There is no assurance that the remaining testicle will produce sperm of adequate quality, and fertility may be affected. Additionally, the remaining testicle is at risk for conditions like testicular torsion or trauma, which could further jeopardize fertility. These risks underscore the importance of considering fertility preservation options before treatment.

The main strengths of this study include its large cohort size and comprehensive follow-up using data from population-based registers. This approach ensures a diverse sample, improving the validity and applicability of the findings while minimizing selection bias compared to earlier studies focused on specific groups (28).

Limitations of this study include the lack of data on the desire to start a family within the full cohort, unsuccessful attempts to conceive naturally that did not result in pursuing fertility treatment, and the presence of female factor infertility, which often plays a large role in couples’ infertility challenges. Brydöy et al., 2005, report that in up to 65% of couples in the general population seeking infertility consultations, female factors play a partial role (29), including female age associated infertility (30). Carson et al. (2021) describe in a large review on Diagnosis and Management of Infertility that the most common causes of infertility are ovulatory dysfunction, male factor infertility, and tubal disease, and that a fairly low percentage, with approximately 1 in 8 women (12.5%) aged 15 to 49 years receive infertility services (31).

When addressing infertility in men with cancer, it’s essential to consider the broader context of female factor infertility, as the two are often interconnected in couples facing these challenges. Women may experience infertility due to cancer or its treatments, especially if they have a history of reproductive cancers such as ovarian or uterine cancer, or if they undergo treatments that impact ovarian function. Additionally, women may face other medical conditions, including poly cystic ovary syndrome, fallopian tube damage, ovulatory disorders or endometriosis, which can complicate their ability to conceive. Lifestyle factors such as overweight or smoking can also have a negative effect on fertility and certain genetic conditions, such Turner syndrome can also play a role in female infertility (32).

For the female partners of male cancer patients who are freezing sperm, a thorough medical evaluation is crucial. This assessment can identify any underlying issues and enhance the chances of future biological parenthood, ensuring both partners are supported in their journey to conceive. Future infertility studies would benefit from including both female and male factor aspects. In the present study we assumed that a history of cancer in the male partner, frequently involving orchiectomy and gonadotoxic therapy, was the main factor contributing to infertility.

In Sweden, treatment of infertility is reimbursed within the tax-funded healthcare services, thus individuals and couples receiving a diagnosis of infertility are currently being offered funded treatment. Therefore, we can assume that the low proportion (18%) of individuals in our cohort undergoing treatment using assisted conception, constituted the true infertile group in the cohort, as they used either cryopreserved or fresh own sperm, or in two cases donor sperm.

Unfortunately, there is a lack of information on individuals comorbidities, lifestyle factors such as smoking and alcohol intake, and details of cancer treatment in the registers used in this study, factors that could have an impact on the final outcome of achieving parenthood or not. Addressing gaps in information on comorbidities among cancer patients who undergo sperm freezing for fertility preservation is essential for improving patient care and outcomes. For future studies, actions to better cover the patients’ health status, could be taken already at referral to the fertility clinic. For instance, standardized health assessments, including smoking and alcohol intake, with detailed health information at the time of sperm cryopreservation, including medical history, current medications, and psychological evaluations should be registered consistently in a medical database, for all patients. This would help identify prevalent comorbidities such as diabetes, cardiovascular issues, and mental health conditions, as well as excessive tobacco and alcohol intake. Also, blood samples should be taken, and in cases with known risk of genetic disorders, genetic testing could be conducted to provide valuable insights.

Another important aspect of addressing comorbidities in fertility preservation is the establishment of interdisciplinary teams. Collaborations among oncologists, reproductive specialists, endocrinologists, and mental health professionals can significantly enhance patient care, ensuring comprehensive support for both cancer treatment and fertility preservation. Also, maintaining an ongoing dialogue between healthcare providers and the patients is crucial, as it helps keep patients informed and engaged throughout the entire process. To further monitor the patients’ health status, fertility treatments and psychological wellbeing, regular follow ups could be of interest to identify trends and correlations.

In our cohort, the majority of the patients had testicular cancer, and previous data indicate, that patients diagnosed with early-stage testicular cancer comprise a relatively healthy group of cancer survivors, demonstrating no increased sick leave compared to individuals without testicular cancer (33). Also, testicular cancer is often diagnosed at Stage 1, when it is confined to one testicle, and there is a high likelihood of maintaining sperm quality after treatment, whether the patient is managed with active surveillance or receives adjuvant therapy following orchiectomy (27). Individuals with more advanced stages of testicular cancer may also resume spermatogenesis and regain normal semen quality after some years (34).

In general, different cancer treatments, drugs and dosages vary in their impact on spermatogenesis. The most common chemotherapeutic treatments for all stages of testicular cancers include a combination of Bleomycin, Etoposide, and Cisplatin (BEP). Carboplatin, taxanes and ifosfamide are other possible options, depending on stage of the disease (35). Cyclophosphamide, commonly used to treat leukemia in pediatric patients, impacts spermatogenesis in a dose-dependent manner (36). The risk of infertility increases with additional treatments for high-risk disease or relapse and rises further when patients undergo conditioning for hematological stem cell transplantation (37). Additionally, retroperitoneal lymph node dissection, a surgical procedure used for some testicular cancer patients, can also result in infertility due to retrograde ejaculation (38). Consequently, the risk of infertility from cancer treatment may encourage patients to choose sperm cryopreservation to increase their chances of biological fatherhood.

Overall, the use of frozen sperm has remained consistently low over the years, ranging from 3% to 11%. This trend is also seen in this cohort, which showed a utilization rate of 6.8%, reflecting findings from other cohort studies (19, 39–45). Several factors can contribute to this low utilization, including the maintenance of natural fertility allowing for natural conception, reluctance to have children, cultural beliefs, and concerns about disease relapse, among others (39, 46). The utilization of frozen sperm in this study may underestimate the actual usage, as the follow-up period is short and utilization is likely to increase as individuals age. Men who cryopreserve their sperm usually do not intend to start a family right after a cancer diagnosis. As their circumstances change and stable relationships form, more individuals may decide to use their frozen sperm in the future (43).

Treatments using IVF/ICSI can also be costly in many countries (47), and individuals may favor insurance-covered options, which often do not include cryopreservation or the ongoing maintenance of frozen samples that require an annual fee. Ethical concerns may also influence decisions, with physicians playing a crucial role in guiding patients through fertility choices (36). Moreover, studies have shown that factors associated with lower likelihood of utilizing cryopreserved sperm include being younger at the time of sperm banking and being diagnosed with testicular cancer (43). Van Casteren et al. (2008) reported in their review on approximately 600 males, a utilization rate of 7,5%, with live births in 49% of the patients (42). In a systematic review by Ferrari et al. (2016) on almost 12,000 patients, the numbers were similar, with approximately 8% of the cancer survivors returning to use their frozen sperm and 49% achieving fatherhood (48). In our study, 37% succeeded in fathering a child through the use of cryopreserved sperm and ART, numbers similar to those presented in previous studies (36, 46, 49). There are also studies presenting even higher numbers, such as Muller et al. (2016), who reported the effectiveness of ART they found that 96 (10.7%) of the patients used their frozen sperm for intrauterine insemination, IVF and ICSI with a cumulative ART success rate of 77% (39). Garcia et al. compared success of ART with cryopreserved sperm in patients with cancer compared with non-cancer individuals, presenting a success rate of 62% in the cancer group and at least the same results in the non-cancer group (19). The changing nature of reproductive decisions emphasizes the importance of studying long term trends in fertility preservation.

While the percentage of cancer survivors returning to utilize their frozen sperm may appear relatively low and relatively stable over the study period, there was a distinct increase in cryopreservation of sperm in 2009 and onwards (**Figure 2**). The rise in cryopreservation may be linked to heightened awareness of fertility preservation among patients and oncologists, following the first international guidelines for fertility preservation provided by ASCO in 2006 (50), but also on an increasing incidence of cancer over time. Depalo et al., 2016, and Muller et al., 2016, have observed comparable temporal patterns (39, 40). The increase in cryopreservation, as illustrated in **Figure 2**, is likely to lead to greater utilization of frozen sperm, ultimately contributing to more successful biological fatherhood in several ways. First, as awareness of the cryopreservation process and its benefits grows, there will likely be increased acceptance of using frozen sperm in ART. This can encourage more men to return to utilize their stored sperm. Additionally, as demand for cryopreservation rises, fertility clinics may expand their services and accessibility for patients. This improvement will enhance support for those using frozen sperm, making the process smoother and more efficient. Moreover, a rise in sperm banking will facilitate valuable data collection regarding the outcomes of using frozen sperm. Such research can validate and enhance ART techniques, ultimately leading to higher success rates in achieving biological fatherhood. Also, as a result of increased utilization of cryopreserved sperm, the use of ICSI is also expected to rise. With high success rates, as demonstrated in our **Supplementary Table S1**, it will likely lead to a higher number of biological fatherhood outcomes.

Previous studies suggest that 15%-30% of male AYA cancer survivors may experience transient infertility lasting for months or even years after cancer cure (51), and in some cases, the initial treatment may shift to a more sterilizing regime. Hence, it’s essential to highlight that sperm preservation can offer these individuals the opportunity to father their own biological children in the future.

Currently, there are few reliable predictors of male fertility after cancer treatment (52), and the permanent lack of spermatogenesis leading to azoospermia after chemotherapy varies widely, from 0% to 63%, depending on diagnosis and treatment regimen (53). Sperm concentration plays a significant role in determining post-treatment sperm parameters and the recovery of spermatogenesis (54). Nevertheless, modern ART techniques have a high success rate, as only a few numbers of functional sperm are needed for successful fertilization (55).

The sperm cryopreservation data is derived from the established regional program for fertility preservation at Karolinska University Hospital, where the service is systematically offered to the entire population within the tax-funded healthcare system. Subsequently, the storage of frozen sperm is free of charge for the patient. Unfortunately, we were not able to explore the cost-effectiveness of sperm cryopreservation in this study. A US study investigating the cost-effectiveness of fertility preservation compared to post therapeutic fertility treatment in testicular cancer patients showed that sperm cryopreservation is the most cost-effective strategy for preserving fertility in men with testicular cancer, the largest patient group in our cohort (56). Cost-effectiveness studies of sperm cryopreservation in a Swedish or Nordic setting would likely be more useful, as the Nordic countries have similar tax-funded healthcare systems. Future studies on this subject are crucial for improving cost-effectiveness in health care planning. The follow-up data for each individual in our cohort were obtained by linking the individuals to their respective data collected in Swedish nationwide population-based registers.

During the study period, cancer treatments have improved, and more people are being diagnosed with cancer while also surviving it. Additionally, there is a shift towards delayed parenthood in Western societies in general.

As many patients are followed for only a short time after treatment, this may result in an underestimation of how many patients actually use their frozen sperm. Though, in **Figure 3**, the cumulative incidence of fatherhood over time since cryopreservation is demonstrated, which is an important indicator reflecting the long-term reproductive outcomes for cancer survivors. By considering the competing risk of death and categorizing results by diagnosis type, we can better understand the nuances of fatherhood in these patients and better aid in optimizing patient care and counseling during a critical time in their lives.

The evolving nature of fertility-related concerns in cancer patients underscores the need to not only assess the impact of cancer treatments on fertility but also recognize the broader societal and demographic factors that influence decisions regarding fertility preservation. When considering fertility preservation, it is essential to understand its significant impact on the patient’s quality of life. For many young cancer patients, one of their primary concerns is the potential effect of chemotherapy and other treatments on their fertility. The ability to have biological children is often a central part of their life plans and sense of identity, making fertility preservation a critical consideration during their treatment process (18, 57, 58). Sperm banking, in particular, provides substantial psychological and emotional support for male cancer patients. It offers them a sense of control over their future, reassurance about their reproductive options, and hope for the possibility of fatherhood, even after cancer treatment (59). These benefits can significantly reduce anxiety and distress during the treatment process and in the years that follow. By preserving the option of future fatherhood, sperm banking helps patients maintain their sense of masculinity and identity, which can be deeply affected by a cancer diagnosis (58). Additionally, sperm banking reduces the risk of long-term emotional distress related to infertility, enabling patients to approach life post-recovery with greater confidence and optimism (60). The emotional advantages of sperm banking are clear, highlighting its importance as a key component of cancer care that supports the overall well-being of male patients (61).

However, not all cancer patients are provided with adequate information about the risk of infertility caused by cancer or its treatment. The lack of information may make patients less likely to consider sperm cryopreservation (59). Therefore, it is crucial that healthcare providers communicate the potential risk of fertility impairment early in the diagnosis and treatment process. Providing timely and comprehensive information on fertility risks ensures that patients have the opportunity to make informed decisions about their reproductive future. Guidelines from organizations such as the American Society of Reproductive Medicine and the American Society of Clinical Oncology recommend that fertility options be discussed with patients as early as possible (7, 8). Patients who are not provided this information are at higher risk of experiencing psychological distress later on (58).

The decisions surrounding fertility preservation are often complex, and a multidisciplinary team approach is essential. Such an approach ensures that patients are properly informed about their options and supported in making the best decisions for their future. It also facilitates the identification of those at greater risk of psychological distress, creating opportunities for ongoing discussions about the psychosocial challenges related to cancer treatment and fertility preservation (58).

Fertility-related concerns for cancer patients often extend beyond their own fertility potential. These concerns may include fears about living long enough to raise children, worries about passing on a genetic predisposition to cancer, and the emotional challenges of disclosing fertility issues to a partner (6). Additionally, as shown in **Supplementary Figure S1**, men who already had children were more likely to become fathers sooner after cryopreservation than those without previous children. This difference could be attributed to a combination of psychological readiness, established relationships, heightened awareness of fertility, support systems, resource availability, and strong motivations for family planning. Understanding these dynamics is important for healthcare providers, as it allows them to better support male patients in their fertility preservation process by tailoring counseling and resources to meet their specific needs.

## 5 Conclusion

Most adolescent and young adult (AYA) cancer survivors who froze their sperm were able to conceive naturally after treatment. However, limited long-term follow-up data prevents definitive conclusions about the long-term use of frozen sperm. Cryopreservation remains a valuable option for those who experience infertility post-treatment, and intracytoplasmic sperm injection (ICSI) is often more effective than traditional IVF.

There are no reliable predictors for testicular toxicity after cancer treatment, and fertility outcomes vary widely among individuals. As cancer treatments evolve, particularly with newer targeted therapies, the long-term impact on fertility remains unclear. To improve fertility preservation strategies, further research is needed to identify which patients are most at risk of infertility and would benefit from early intervention. Enhanced longitudinal studies and the development of predictive models, informed by evolving treatment protocols, are essential to address current research gaps and guide future investigations in this area.

## Data Availability

The raw data supporting the conclusions of this article will be made available by the authors, without undue reservation.

## Supplementary material

Supplementary Figure S1 Cumulative incidence of fatherhood after cancer by years since diagnosis in men with and without previous children, with death as a competing risk.

Supplementary Figure S2 Crude probability of fatherhood after cancer by years since diagnosis and age-group at diagnosis.

Supplementary Table S2 Hazard ratios of fathering a child after cancer. *Other hematological tumors include chronic myeloid leukemia, myeloma and myelodysplastic syndrome. **Other solid tumors include testicular cancer of unspecified type, malignant melanoma and tumors of the lung, digestive tract, head and neck.
