# Supplementary material for: Parenthood in a Swedish prospective cohort of 1,378 adolescents and young adults banking semen for fertility preservation at time of cancer diagnosis
Source: Front Endocrinol (Lausanne). 2024 Dec 10;15:1502479. doi: 10.3389/fendo.2024.1502479 (PMC11667001; doi:10.3389/fendo.2024.1502479)
Supplement: Supplementary file 1 [file Table1.docx]

**Supplementary table 1.** Method of successful IVF/ICSI, 2007-2020

| ART | Fresh | Frozen | Unknown  (frozen/fresh) | Total |
| --- | --- | --- | --- | --- |
| ICSI, N (%) | 33 (48) | 25 (37) | 10 (15) | 68 |
| IVF , N (%) | 4 (40) | 3 (30) | 3 (30) | 10 |
| Donated, N (%) | 0 | 2 (100) | 0 | 2 |
| Total, N (%) | 37 (46) | 30 (38) | 13 (16) | 80 |
