# Supplementary figures and images for: Parenthood in a Swedish prospective cohort of 1,378 adolescents and young adults banking semen for fertility preservation at time of cancer diagnosis
Source: Front Endocrinol (Lausanne). 2024 Dec 10;15:1502479. doi: 10.3389/fendo.2024.1502479 (PMC11667001; doi:10.3389/fendo.2024.1502479)

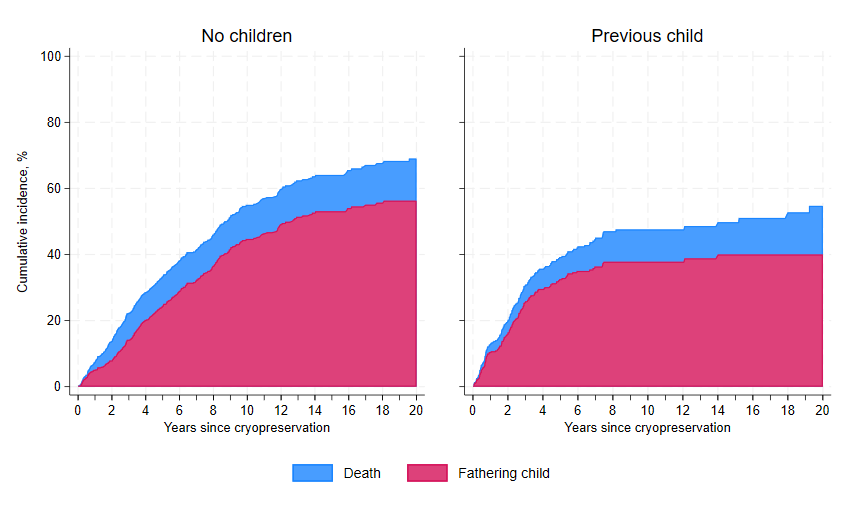

Supplement: Supplementary Figure S1 — Cumulative incidence of fatherhood after cancer by years since diagnosis in men with and without previous children, with death as a competing risk. [file Image1.tif]

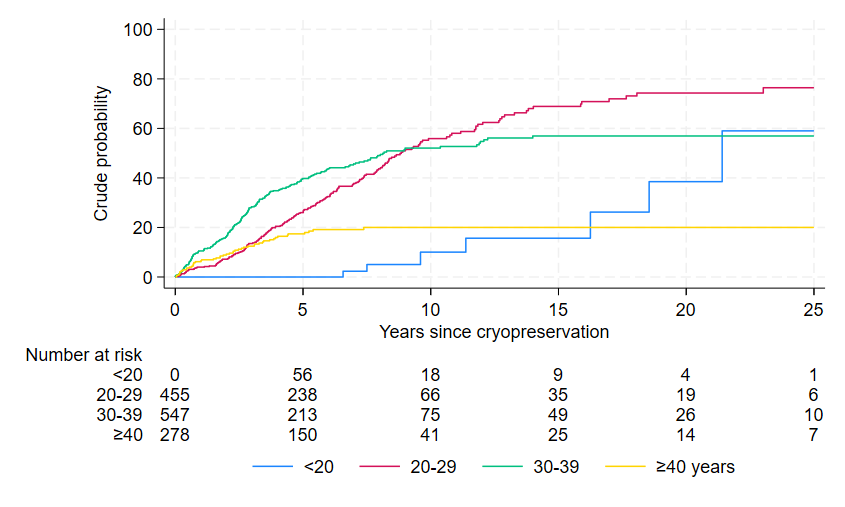

Supplement: Supplementary Figure S2 — Crude probability of fatherhood after cancer by years since diagnosis and age-group at diagnosis. [file Image2.tif]
