# Supplementary material for: Parenthood in a Swedish prospective cohort of 1,378 adolescents and young adults banking semen for fertility preservation at time of cancer diagnosis
Source: Front Endocrinol (Lausanne). 2024 Dec 10;15:1502479. doi: 10.3389/fendo.2024.1502479 (PMC11667001; doi:10.3389/fendo.2024.1502479)
Supplement: Supplementary Table S2 — Hazard ratios of fathering a child after cancer. *Other hematological tumors include chronic myeloid leukemia, myeloma and myelodysplastic syndrome. **Other solid tumors include testicular cancer of unspecified type, malignant melanoma and tumors of the lung, digestive tract, head and neck. [file Table2.docx]

**Supplemental table 2.** Hazard ratios of fathering a child after cancer

|  | Univariable model | | Multivariable adjusted model | |
| --- | --- | --- | --- | --- |
|  | HR | 95% CI | HR | 95% CI |
| Diagnosis |  |  |  |  |
| Testicular seminoma | 1.00 | Ref | 1.00 | Ref |
| Testicular non seminoma | 1.01 | 0.79-1.28 | 1.02 | 0.80-1.31 |
| Hodgkin lymphoma | 0.86 | 0.63-1.18 | 0.85 | 0.62-1.17 |
| Non Hodgkin lymphoma | 0.91 | 0.65-1.27 | 0.91 | 0.65-1.28 |
| Acute leukemia | 0.70 | 0.44-1.12 | 0.79 | 0.49-1.27 |
| Other hematological* | 0.47 | 0.25-0.89 | 0.54 | 0.28-1.02 |
| Prostate | 0.42 | 0.23-0.76 | 0.76 | 0.41-1.42 |
| Colorectal | 1.01 | 0.59-1.72 | 1.30 | 0.75-2.24 |
| CNS | 0.45 | 0.19-1.11 | 0.55 | 0.22-1.36 |
| Sarcoma | 1.48 | 0.88-2.49 | 1.53 | 0.91-2.59 |
| Other solid tumors** | 1.08 | 0.75-1.56 | 1.19 | 0.82-1.72 |
| Fatherhood at cryopreservation |  |  |  |  |
| No |  |  | 1.00 | Ref |
| Yes |  |  | 1.23 | 1.00-1.52 |
| Calendar year at cryopreservation |  |  |  |  |
| 1988-1999 |  |  | 1.00 | Ref |
| 2000-2004 |  |  | 0.87 | 0.61-1.24 |
| 2005-2009 |  |  | 1.20 | 0.87-1.66 |
| 2010-2014 |  |  | 1.11 | 0.83-1.50 |
| 2015-2020 |  |  | 0.83 | 0.61-1.15 |
| Attained age |  |  |  |  |
| 20-29 years |  |  | 1.00 | Ref |
| 30-39 years |  |  | 2.54 | 1.96-3.30 |
| ≥40 years |  |  | 0.84 | 0.59-1.20 |

*Other hematological tumors include chronic myeloid leukemia, myleoma and myelodysplastic syndrome.
**Other solid tumors include testicular cancer of unspecified type, malignant melanoma and tumors of the lung, digestive tract, head and neck.
